# Weaning performance prediction in lactating sows using machine learning, for precision nutrition and intelligent feeding

**DOI:** 10.1016/j.aninu.2025.01.007

**Published:** 2025-04-01

**Authors:** Jiayi Su, Xiangfeng Kong, Wenliang Wang, Qian Xie, Chengming Wang, Bie Tan, Jing Wang

**Affiliations:** a Key Laboratory of Hunan Province for the Products Quality Regulation of Livestock and Poultry, College of Animal Science and Technology, Hunan Agricultural University, Changsha 410128, China; b Laboratory of Animal Nutritional Physiology and Metabolic Process, Key Laboratory of Agro-Ecological Processes in Subtropical Region, National Engineering Laboratory for Pollution Control and Waste Utilization in Livestock and Poultry Production, Institute of Subtropical Agriculture, Chinese Academy of Sciences, Changsha 410125, China; c Yuelushan Laboratory, Changsha 410128, China

**Keywords:** Weaning performance prediction, Lactating sow, Feature selection, Machine learning, Precision feeding

## Abstract

Traditional feeding strategy during lactation can result in nutrient deficiencies and negatively impact long-term productivity, compromising both the sustainability and profitability of the swine industry. Precision feeding, supported by decision-making systems built on advanced predictive models, offers a promising solution to address these challenges. This study aimed to develop prediction models for weaning performance, focusing on key indicators such as weaned litter weight (WLW), weaned litter size (WLS), dry matter in milk (DMm), and nitrogen in milk (Nm). The models integrate farm management practices and feed nutrient composition, providing a data-driven framework for optimizing performance. A total of 10,089 observations were collected from 17 trial pig farms across eight provinces in China. Eleven statistical and machine learning (ML) regression algorithms were employed, incorporating stratified sampling and the recursive feature elimination method for feature selection. The findings demonstrated that the ensemble learning models, specifically random forest and gradient boosting decision tree regression, delivered the best overall performance, with a coefficient of determination (*R*^2^) ranging from 0.40 to 0.80 and a mean absolute error (MAE) between 0.11 and 4.36. The shapley additive explanations (SHAP) heatmap used for feature importance analysis revealed that, although the key predictors of weaning performance varied across models, this study newly identified lactation duration, birth litter weight, parity, and backfat thickness on the 7th day of lactation (L.d7BF) as consistently important features across different models. The discrepancies between correlation analysis and feature importance suggest the presence of non-linear relationships, feature interactions, and multicollinearity within the dataset. This study presents a novel framework that provides valuable insights into the factors influencing weaning performance under diverse management practices and feed nutrient conditions. The optimized prediction model can be employed to guide real-time sensor-based precision feeding systems, thereby enhancing efficiency and sustainability in swine production.

## 1 Introduction

The efficient and sustainable development of the swine industry is crucial for meeting human meat demands and ensuring food safety (Tian and von Cramon-Taubadel, 2020). The reproductive capacity of sows lies at the heart of the swine industry. In lactating sows, high milk production combined with low voluntary feed intake often results in nutrient deficiencies, which can negatively impact their lifelong reproductive performance (Noblet and Etienne, 1989). Conventional feeding strategy during lactation is based on a close to ad libitum delivery of a single diet (Gauthier et al., 2022a). Over time, these nutritional imbalances can lead to long-term declines in sow productivity, affecting the sustainability and profitability of swine production.

Precision feeding (PF), as an innovative feeding strategy, holds great promise for delivering the right amount of nutrients at the right time for each animal (Gauthier et al., 2022a). This strategy relies on smart devices or decision systems with advanced models for tailored nutrition. However, limited adoption of sensor technology due to cost and technical challenges, along with the difficulty of measuring sow body weight—a key variable, restricts data collection and hinders model development and precision feeding (Su et al., 2024). The weaning performance is usually used as proxies for milk production, which could estimate the nutrient output in milk and the variable nutrient requirements of individuals at a lower expense (Quesnel et al., 2015). Thus, constructing models for weaning performance in lactating sows is the most practical approach to assess feeding strategies and a key step toward developing decision support systems for precision feeding (Gauthier et al., 2022b). This enables progress despite limited real-time data and supports better feeding decisions in practical conditions.

However, numerous factors affect weaning performance, such as genetic factor, parity of sows, body weight and backfat thickness at farrowing, litter size, nutrient intake during the lactation period, and environmental factors including temperature and humidity (Gaillard et al., 2021). Selecting key factors helps to reduce computation time, enhance prediction accuracy, and improve the interpretability of both the data and results (Li et al., 2017). Therefore, this study aims to identify the machine learning (ML) algorithm that yields the best performance for predicting weaning performance and determine the most contributing factors.

## 2 Materials and methods

### 2.1 Description of study area

The study encompassed 17 trial pig farms, comprising 10,089 observations across 8 provinces in China: Beijing, Hebei, Anhui, Hubei, Jiangxi, Hunan, Sichuan, and Yunnan. These farms implemented diverse management practices and distinct nutritional programs, providing a comprehensive representation of varying production conditions.

### 2.2 Data acquisition

#### 2.2.1 Basic information of sows

Understanding and accounting for the basic information of sows are essential for the accurate prediction and effective management of weaning performance. Basic information included parity of sows, gestation days, region and strain of sows (RSS), breed of sows, strain and breed of boars. For convenience, categorical features, such as strain and breed of boars, breed, and RSS, are represented numerically.

The RSS categories were labeled as follows: New American = 1, American = 2, Canadian = 3, French = 4, and Danish = 5. For breed, the labels were: Landrace × Yorkshire = 1, Landrace = 2, Yorkshire = 3, Duroc = 4, Yorkshire × Landrace = 5, and Pietrain = 6. The strain and breed of boars were represented by the following numeric labels: American Yorkshire = 1, American Landrace = 2, New American Landrace = 3, New American Yorkshire = 4, New American Duroc = 5, American Duroc = 6, American Pietrain = 7, French Landrace = 8, French Yorkshire = 9, Danish Landrace = 10, Canadian Landrace = 11, Canadian Yorkshire = 12, and Canadian Duroc = 13.

#### 2.2.2 Environmental information

Temperature and humidity fluctuations in the pig barn during the lactation period can significantly affect the weaning performance of sows. For this experiment, the selected environmental factors included temperature and humidity measured at different stages: days 1, 7, and 21 of lactation. The data were collected using temperature and humidity sensors to ensure accurate monitoring.

#### 2.2.3 Sow body condition management and nutritional level of feed

In lactating sows, priority is given to maintenance and milk production. Body reserves often contribute to the supply of these priority functions (Dourmad et al., 2008). Therefore, monitoring backfat thickness is essential to assess whether feeding strategies are optimal for reproductive performance (Choi et al., 2019). In this experiment, backfat thickness on days 1, 7, and 21 of lactation were measured. The measurements were typically taken at the last rib (P2), located 6.5 cm from the midline over the last rib, using an A mode ultrasound scanner (PIGLOG105, SFK LEBLANC, Kolding, Region of Southern Denmark, North Denmark Region, Denmark) (Sulabo et al., 2010).

Nutrient requirements for sows are relatively variable throughout lactation (NRC, 2012). It is recommended to align nutritional supplies with the sows' requirements to maximize milk production and piglet growth, while minimizing reproductive issues in sows after weaning (Dourmad et al., 2008). Feeding systems during lactation are based on a close to ad libitum delivery of a single diet. So, in this experiment, the nutritional level in the feed were determined, including dry matter (DM, method 2001.12), ash (method 942.05), crude protein (CP, method 954.01), ether extract (EE, method 920.39), phosphorus (P, method 965.17), calcium (Ca, method 927.02), neutral detergent fiber (NDF, method 2002.04), acid detergent fiber (ADF, method 973.18), soluble dietary fiber (SDF, method 993.19), insoluble dietary fiber (IDF, method 991.42), and total dietary fiber (TDF, method 992.16) according to the procedures of AOAC (AOAC, 2023). The ratio of IDF/SDF and metabolizable energy (ME) were calculated (Kil et al., 2013).

#### 2.2.4 Weaning performance criteria and nutrient output in milk

The target variables included weaned litter weight (WLW), weaned litter size (WLS), and the calculated nutrient requirements for milk production—specifically, dry matter in milk (DMm) and nitrogen in milk (Nm)—based on other performance criteria in lactating sows. The litter weight gain (LWG) was computed as the difference between WLW and the birth litter weight (BLW). The litter average daily gain (LADG) was computed as LWG divided by the duration of lactation. The piglet average weight gain (PADG) was computed as LADG divided by WLS (Gauthier et al., 2022b). DMm and Nm were computed according to the equation (Noblet and Etienne, 1989):

DMm(kg/d)=(0.72×PADG×WLS−7×WLS)/1000;

Nm(g/d)=0.0257×PADG×WLS+0.42×WLS.

### 2.3 Data preparation and preliminary analysis

Cleaning steps of the database were performed to remove uncommon observations from the database. Prior to training, 3 categorical features were transformed into dummy variables (Amoukou et al., 2021). After encoding, if a sow's RSS was New American, its corresponding dummy variables were RSS_1 = 1, RSS_2 = 0, RSS_3 = 0, RSS_4 = 0, RSS_5 = 0. If a sow's RSS was Canadian, its corresponding dummy variables were RSS_1 = 0, RSS_2 = 0, RSS_3 = 1, RSS_4 = 0, RSS_5 = 0. If a sow's breed was Landrace × Yorkshire, its corresponding dummy variables were Breed_1 = 1, Breed_2 = 0, Breed_3 = 0, Breed_4 = 0, Breed_5 = 0, Breed_6 = 0. If a sow's breed was Yorkshire, its corresponding dummy variables were Breed_1 = 0, Breed_2 = 0, Breed_3 = 1, Breed_4 = 0, Breed_5 = 0, Breed_6 = 0. If the strain and breed of boars was American Yorkshire, its corresponding dummy variables were Strain and breed of boars_1 = 1, Strain and breed of boars_2 = 0, Strain and breed of boars_3 = 0, Strain and breed of boars_4 = 0, Strain and breed of boars_5 = 0, Strain and breed of boars_6 = 0, Strain and breed of boars_7 = 0, Strain and breed of boars_8 = 0, Strain and breed of boars_9 = 0, Strain and breed of boars_10 = 0, Strain and breed of boars_11 = 0, Strain and breed of boars_12 = 0, Strain and breed of boars_13 = 0. If the strain and breed of boars was Danish Landrace, its corresponding dummy variables were Strain and breed of boars_1 = 0, Strain and breed of boars_2 = 0, Strain and breed of boars_3 = 0, Strain and breed of boars_4 = 0, Strain and breed of boars_5 = 0, Strain and breed of boars_6 = 0, Strain and breed of boars_7 = 0, Strain and breed of boars_8 = 0, Strain and breed of boars_9 = 0, Strain and breed of boars_10 = 1, Strain and breed of boars_11 = 0, Strain and breed of boars_12 = 0, Strain and breed of boars_13 = 0.

In this study, the distributions of four target variables across different provinces were visualized using the boxplot generated with the seaborn library in Python (Aslam et al., 2023). Additionally, descriptive statistics including mean, standard deviation (SD), coefficient of variation (CV), minimum, the 25th percentile, the 50th percentile, the 75th percentile, and maximum were calculated using the describe function from the Pandas library in Python. Pairwise Spearman correlation coefficients among features (excluding categorical features) and target variables were computed using the corr method in Pandas library (Gupta and Bagchi, 2024).

### 2.4 ML statistical analysis

#### 2.4.1 Feature selection techniques

Feature selection techniques aim at identifying and selecting the most relevant features from the dataset to improve model performance and reduce dimensionality. In this study, features were chosen using recursive feature elimination (RFE), a technique based on the Random Forest Regressor, which iteratively evaluates decreasing subsets of features. The parameter n.features.to.select was validated by recursive feature elimination with cross-validation (RFECV), enabling the selection of the optimal number of features through cross-validation (Ruan et al., 2022).

#### 2.4.2 ML algorithms

**Fig. 1:**
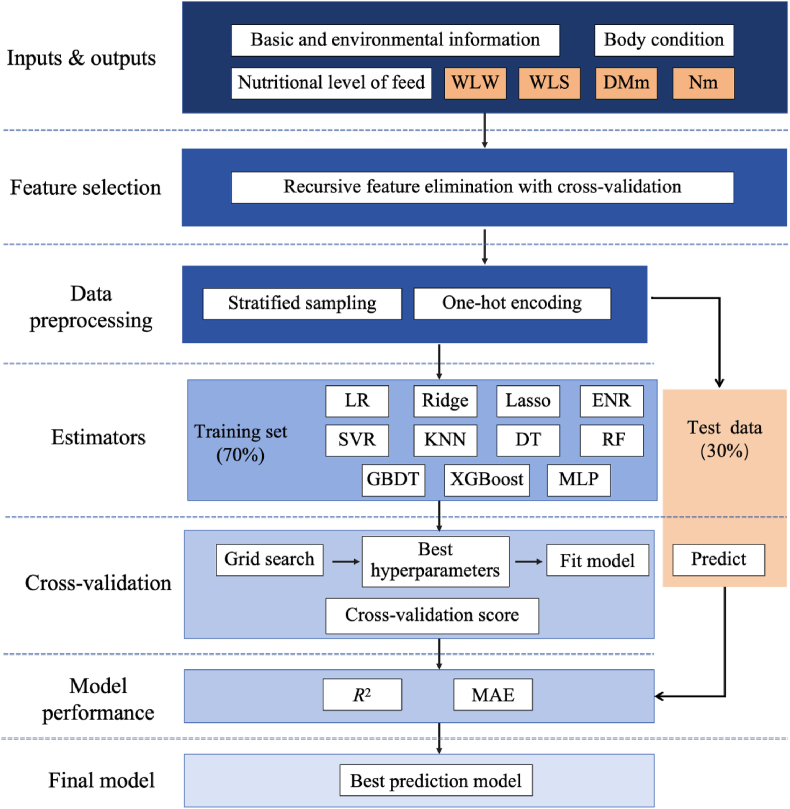
The workflow of the machine learning-driven prediction. WLW = weaned litter weight; WLS = weaned litter size; DMm = dry matter in milk; Nm = nitrogen in milk; LR = linear regression; Ridge = ridge regression; Lasso = lasso regression; ENR = ElasticNet regression; SVR = support vector regression; KNN = k-nearest neighbors; DT = decision tree; RF = random forest; GBDT = gradient boosting decision tree; XGBoost = extreme gradient boosting; MLP = multi-layer perceptron; *R*^2^ = coefficient of determination; MAE = mean absolute error.

All ML algorithms were completed using the Anaconda Navigator (version 2.6.0, Anaconda Inc., Austin, TX, USA) based on Python (version 3.12, Python Software Foundation, Wilmington, DE, USA). The StratifiedShuffleSplit package was used to split training data and test data (70%: 30%). The workflow of the prediction process is illustrated in Fig. 1.

ML models (also referred to as estimators) were fitted to the dataset using Scikit-learn, an open-source machine learning library that supports supervised learning. Eleven different algorithms were evaluated, including linear regression (LR), ridge regression (Ridge), lasso regression (Lasso), elastic net regression (ENR), support vector regression (SVR), k-nearest neighbors (KNN), decision tree (DT), random forest (RF), gradient boosting decision tree (GBDT), extreme gradient boosting (XGBoost), and multi-layer perceptron (MLP). The specific descriptions of these algorithms are presented in Table 1. Hyperparameters for each model (Table 1) were optimized using 5-fold cross-validation via the GridSearchCV package, and the best hyperparameters were used to fit the models.

**Table 1:** Specific estimator descriptions and parameter grids for tuning hyperparameters.

Estimators	Description	Grids
Linear regression	Fits a straight line or surface that minimizes the discrepancies between predicted and actual output values.	fit.intercept: [True, False]
Ridge regression	Employs L2 regularization to reduce errors caused by overfitting on training data.	alpha: [0.1, 0.5, 1.0]
Lasso regression	Employs L1 regularization to reduce errors caused by overfitting on training data.	alpha: [0.1, 0.5, 1.0]
ElasticNet regression	A regularized regression method that linearly combines the L1 and L2 penalties of the lasso and ridge methods.	alpha: [0.1, 0.5, 1.0], l1.ratio: [0.1, 0.5, 0.7]
Support vector regression	Optimizes a function by finding a tube that approximates a continuous-valued function while minimizing the prediction error.	kernel: [‘linear’, ‘poly’, ‘rbf’], C: [0.1, 1, 10]}
k-Nearest neighbors	Uses proximity to make predictions about the grouping of an individual data point.	n.neighbors: [3, 5, 7]
Decision tree	Employs a divide and conquer strategy by conducting a greedy search to identify the optimal split points within a tree.	max.depth: [None, 5, 10]
Random forest	Creates a multitude of decision trees during training. The output is the average of the predictions of the trees.	n.estimators: [100, 200, 300], max.depth: [None, 5, 10]
Gradient boosting decision tree	Bases on boosting in a functional space, where the target is pseudo-residuals instead of residuals as in traditional boosting.	n.estimators: [100, 200, 300], max.depth: [3, 5, 10]
Extreme gradient boosting	An optimized distributed gradient boosting library designed to be highly efficient, flexible and portable.	n.estimators': [100, 200, 300], max.depth: [3, 5, 10]
Multilayer perceptron	A modern feedforward artificial neural network, consisting of fully connected neurons with a nonlinear activation function.	hidden.layer.sizes: [(50), (100), (50, 50)], activation: [‘relu’, ‘tanh’]

All random.state values used in this analysis were set to 42.

#### 2.4.3 Evaluation metrics

The coefficient of determination (*R*^2^) and mean absolute error (MAE) between the predicted target variables and the observed target variables were used for model performance evaluation. MAE is a performance metric used to evaluate regression models. MAE measures the average absolute difference between the predicted values and the actual values. The small value of MAE indicates that the prediction model has better performance (Hodson, 2022). *R*^2^ is a metric used to evaluate the goodness of fit of a regression model. *R*^2^ measures the proportion of the variance in the target variable that can be explained by the model. Its value varies between 0 and 1. The *R*^2^ value approaching 1 is an indication of better performance (Tatachar, 2021).

Firstly, the robustness and generalization of models were assessed through 5-fold cross-validation utilizing the cross.val.score package on the training data. Subsequently, the identical evaluation approach was applied to the test data.

#### 2.4.4 Model interpretation

In the pursuit of transparent ML, explanations are as vital as model construction for decision support (Martini et al., 2022). The shapley additive explanations (SHAP) framework was employed to interpret predictions generated by complex black box ML algorithms (Lundberg et al., 2020). SHAP analysis is favored due to its local accuracy, consistency, and ability to handle missing values (Yao et al., 2023). Here, the SHAP heatmap was utilized which is one of the key visualization tools (Moncada-Torres et al., 2021).

## 3 Results

### 3.1 Data exploration

#### 3.1.1 Statistics of weaning performance and its influencing factor variables

Table 2 presents descriptive statistics for various features related to the dataset, including their mean, SD, CV, and percentiles (minimum, the 25th, 50th, 75th, and maximum). The mean parity was 3.767, with a wide range from 1 to 15. Gestation days averaged 115.633 days, with minimal variation. Temperatures and humidity levels showed moderate variation, with CVs ranging from 0.067 to 0.098. BLW and litter size displayed substantial variability, indicated by high CVs (0.256 and 0.243, respectively). The duration of lactation averaged 21.838 days, with a CV of 0.076. WLS and WLW also exhibited variation. DMm and Nm had CVs of 0.217 and 0.197, respectively.

**Table 2:** Descriptive statistics of weaned performance and its influencing factors for each variable.

Feature name	Mean	SD	CV	Min	The 25th percentile	The 50th percentile	The 75th percentile	Max
Parity	3.767	2.626	0.697	1.000	2.000	3.000	5.000	15.000
Gestation days, d	115.633	1.519	0.013	101.000	115.000	116.000	116.000	125.000
L.d1T, °C	24.581	2.403	0.098	18.000	23.000	26.000	26.000	28.000
L.d7T, °C	24.062	2.144	0.089	18.000	23.000	24.000	24.000	29.000
L.d21T, °C	24.593	2.142	0.087	16.000	23.000	25.000	25.000	29.000
L.d1H, %	58.379	3.930	0.067	37.000	56.000	58.000	61.000	68.000
L.d7H, %	56.873	5.022	0.088	23.000	53.000	59.000	59.000	65.000
L.d21H, %	57.102	5.382	0.094	23.000	53.000	60.000	60.000	70.000
L.d1BF, mm	17.237	2.277	0.132	8.000	16.000	18.000	19.000	35.000
L.d7BF, mm	16.575	1.667	0.101	8.000	15.000	17.000	18.000	26.000
L.d21BF, mm	15.244	1.315	0.086	8.000	14.000	15.000	16.000	24.000
Birth litter weight, kg	16.287	4.166	0.256	4.300	13.500	16.500	19.360	28.150
Litter size	11.885	2.884	0.243	1.000	10.000	12.000	14.000	25.000
Duration of lactation, d	21.838	1.670	0.076	14.000	21.000	21.000	23.000	34.000
WLS	10.710	1.258	0.117	2.000	10.000	11.000	11.000	19.000
WLW, kg	70.636	7.396	0.105	50.780	66.000	69.200	75.400	91.250
DMm, kg/d	1.737	0.377	0.217	0.750	1.450	1.720	1.980	3.390
Nm, g/d	69.188	13.596	0.197	32.260	59.170	68.360	77.980	129.170

SD = standard deviation; CV = coefficient of variation; Min = minimum; Max = maximum; L.d1T = temperature on the 1st day of lactation; L.d7T = temperature on the 7th day of lactation; L.d21T = temperature on the 21st day of lactation; L.d1H = humidity on the 1st day of lactation; L.d7H = humidity on the 7th day of lactation; L.d21H = humidity on the 21st day of lactation; L.d1BF = backfat thickness on the 1st day of lactation; L.d7BF = backfat thickness on the 1st day of lactation; L.d21BF = backfat thickness on the 1st day of lactation; WLS = weaned litter size; WLW = weaned litter weight; DMm = dry matter in milk; Nm = nitrogen in milk.

#### 3.1.2 The nutritional levels of lactation diets in different pig farms

Table 3 shows that the nutritional levels of lactation feed varied across different pig farms. The DM content ranged from 872.00 to 914.00 g/kg, ash content ranged from 33.00 to 64.00 g/kg DM, CP content ranged from 133.00 to 178.00 g/kg DM, and EE content ranged from 34.00 to 89.00 g/kg DM, indicating variability in DM, ash, CP, and EE contents. The P content ranged from 3.00 to 13.00 g/kg DM, Ca content ranged from 6.00 to 11.00 g/kg DM, and ME ranged from 11.66 to 13.51 MJ/kg DM, reflecting differences in P, Ca, and ME levels. ADF content ranged from 34.00 to 76.00 g/kg DM, NDF content ranged from 54.00 to 204.00 g/kg DM, SDF content ranged from 10.00 to 34.00 g/kg DM, IDF content ranged from 51.00 to 180.00 g/kg DM, and TDF content ranged from 61.00 to 213.00 g/kg DM, showing differences in ADF, NDF, SDF, IDF, and TDF.

**Table 3:** The analyzed chemical composition of lactation diets in different pig farms.

Farm	DM, g/kg	Ash, g/kg DM	CP, g/kg DM	EE, g/kg DM	P, g/kg DM	Ca, g/kg DM	ME, MJ/kg DM	ADF, g/kg DM	NDF, g/kg DM	SDF, g/kg DM	IDF, g/kg DM	IDF/SDF	TDF, g/kg DM
A	886.00	52.00	135.00	37.00	3.00	6.00	12.24	52.00	204.00	34.00	180.00	5.29	213.00
B	885.00	57.00	178.00	89.00	7.00	9.00	12.32	74.00	122.00	22.00	96.00	4.36	118.00
C	899.00	51.00	165.00	34.00	5.00	6.00	12.43	43.00	155.00	29.00	105.00	3.62	134.00
D	896.00	59.00	160.00	50.00	6.00	7.00	12.81	35.00	119.00	10.00	101.00	10.10	111.00
E	883.00	55.00	175.00	48.00	8.00	10.00	13.51	64.00	113.00	18.00	90.00	5.00	108.00
F	884.00	53.00	167.00	50.00	7.00	9.00	13.18	61.00	114.00	21.00	90.00	4.29	111.00
G	883.00	55.00	175.00	48.00	8.00	10.00	13.51	64.00	113.00	18.00	90.00	5.00	108.00
H	887.00	53.00	172.00	73.00	6.00	7.00	12.19	34.00	54.00	25.00	105.00	4.20	130.00
I	885.00	53.00	175.00	60.00	10.00	9.00	12.18	76.00	95.00	18.00	81.00	4.50	99.00
J	888.00	33.00	171.00	60.00	7.00	8.00	11.88	49.00	167.00	20.00	134.00	6.70	154.00
K	878.00	33.00	133.00	62.00	3.00	8.00	11.66	67.00	123.00	15.00	66.00	4.40	81.00
L	878.00	33.00	133.00	62.00	3.00	8.00	11.66	67.00	123.00	15.00	66.00	4.40	81.00
M	887.00	54.00	169.00	89.00	11.00	7.00	12.39	68.00	132.00	21.00	100.00	4.76	121.00
N	883.00	58.00	160.00	72.00	12.00	9.00	11.97	48.00	120.00	10.00	92.00	9.20	102.00
O	872.00	51.00	165.00	63.00	6.00	8.00	13.50	60.00	109.00	19.00	88.00	4.63	107.00
P	914.00	64.00	167.00	86.00	13.00	11.00	12.33	35.00	83.00	10.00	51.00	5.10	61.00
Q	914.00	64.00	167.00	86.00	13.00	11.00	12.33	35.00	83.00	10.00	51.00	5.10	61.00

DM = dry matter; CP = crude protein; EE = ether extract; P = phosphorus; Ca = calcium; ME = metabolizable energy; ADF = acid detergent fiber; NDF = neutral detergent fiber; SDF = soluble dietary fiber; IDF = insoluble dietary fiber; TDF = total dietary fiber.

#### 3.1.3 Preliminary analysis of target variables in different provinces

**Fig. 2:**
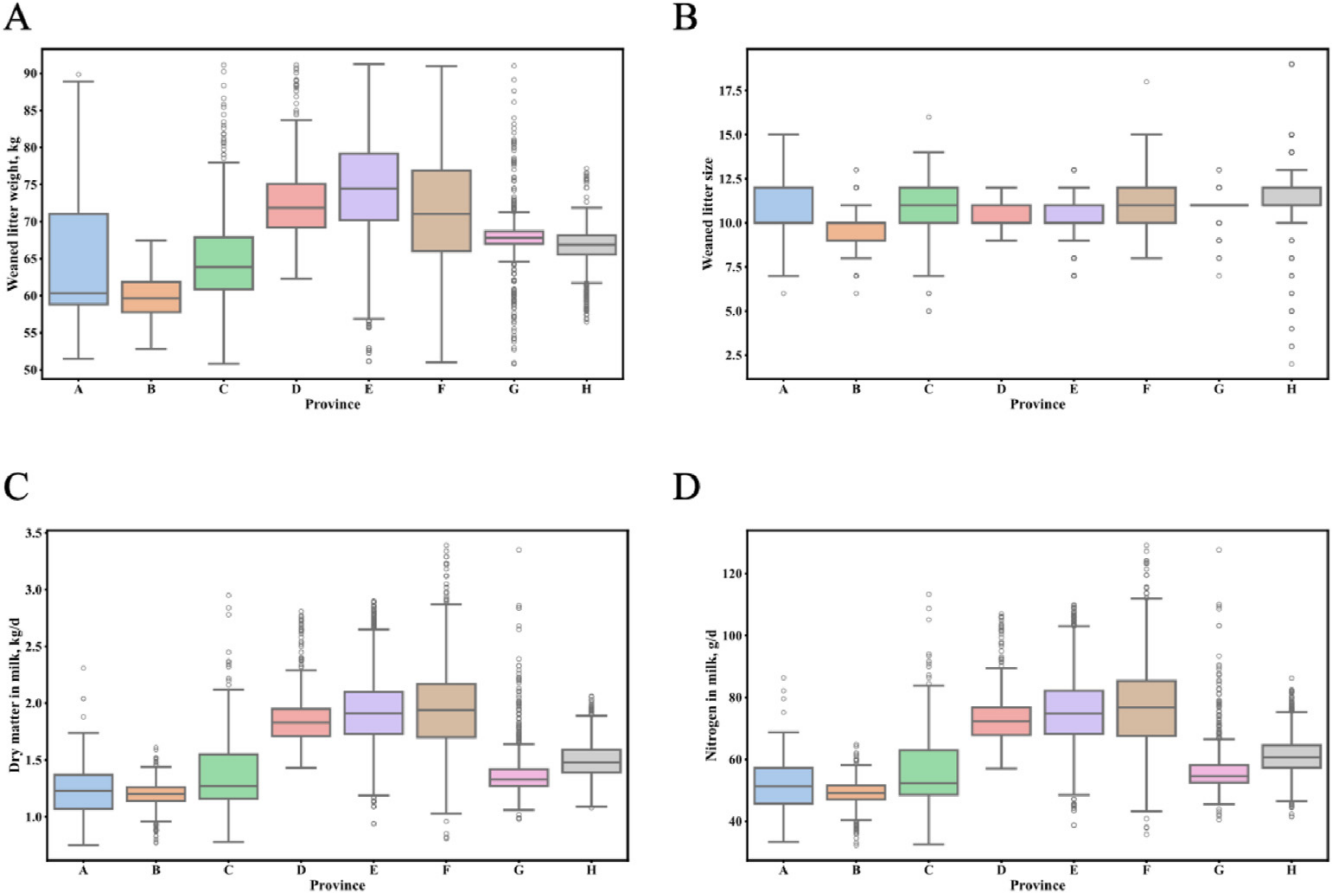
Preliminary analysis of WLW, WLS, DMm, and Nm in different provinces. (A) WLW. (B) WLS. (C) DMm. (D) Nm. WLW = weaned litter weight; WLS = weaned litter size; DMm = dry matter in milk; Nm = nitrogen in milk. A represents Beijing; B represents Hebei; C represents Anhui; D represents Hubei; E represents Jiangxi; F represents Hunan; G represents Sichuan; H represents Yunnan. The boxplot displays the lower, median, and upper quartiles of the data, while the whiskers extend to the minimum and maximum values. Any points outside the whiskers represent outliers.

Figure 2 illustrates notable variations in the minimum, the 25th percentile, median, the 75th percentile, and maximum values of the four target variables across the eight provinces. Therefore, stratified sampling was ultimately chosen for modeling.

#### 3.1.4 Correlation analysis

**Fig. 3:**
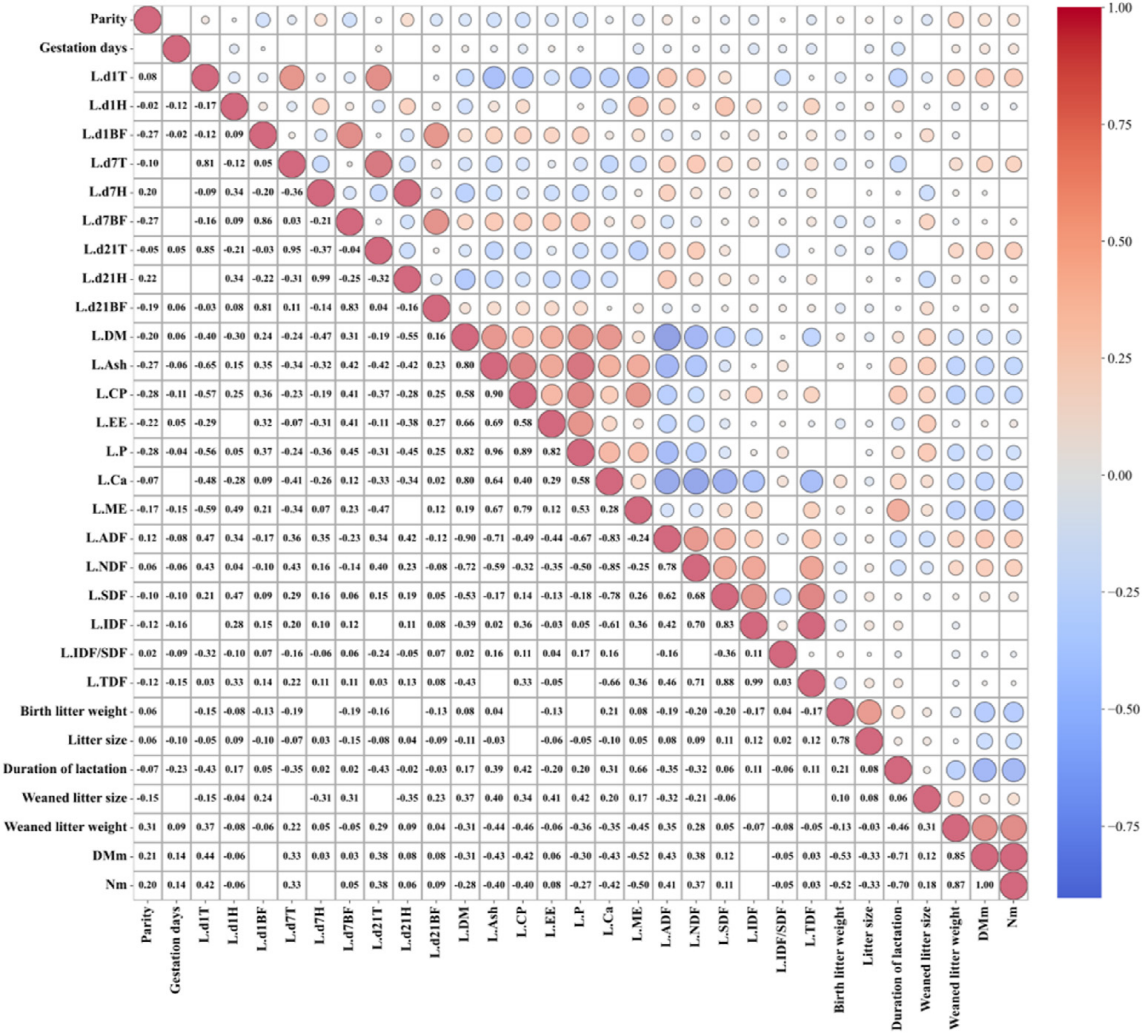
Spearman correlation coefficient heatmap based on features (excluding categorical features) and target variables. The blank indicates that the correlation is not significant (*P* > 0.05). L.d1T = temperature on the 1st day of lactation; L.d7T = temperature on the 7th day of lactation; L.d21T = temperature on the 21st day of lactation; L.d1H = humidity on the 1st day of lactation; L.d7H = humidity on the 7th day of lactation; L.d21H = humidity on the 21st day of lactation; L.d1BF = backfat thickness on the 1st day of lactation; L.d7BF = backfat thickness on the 1st day of lactation; L.d21BF = backfat thickness on the 1st day of lactation; RSS = region and strain of sows; L.DM = dry matter content of lactation feed; L.Ash = ash content of lactation feed; L.CP = crude protein content of lactation feed; L.EE = ether extract content of lactation feed; L.P = phosphorus content of lactation feed; L.Ca = calcium content of lactation feed; L.NDF = neutral detergent fiber content of lactation feed; L.ADF = acid detergent fiber content of lactation feed; L.IDF = insoluble dietary fiber content of lactation feed; L.SDF = soluble dietary content of lactation feed; L.IDF/SDF = the ratio of IDF to SDF content of lactation feed; L.TDF = total dietary fiber content of lactation feed; L.ME = metabolizable energy content of lactation feed; WLW = weaned litter weight; WLS = weaned litter size; DMm = dry matter in milk; Nm = nitrogen in milk.

The Spearman correlation analysis findings for features (excluding categorical features) are illustrated in Fig. 3. WLW had negative correlations with humidity on the 1st day of lactation (L.d1H), backfat thickness on the 1st day of lactation (L.d1BF), backfat thickness on the 7th day of lactation (L.d7BF), DM content of lactation feed (L.DM), ash content of lactation feed (L.Ash), CP content of lactation feed (L.CP), EE content of lactation feed (L.EE), P content of lactation feed (L.P), Ca content of lactation feed (L.Ca), ME content of lactation feed (L.ME), IDF content of lactation feed (L.IDF), the ratio of IDF to SDF content of lactation feed (L.IDF/SDF), TDF content of lactation feed (L.TDF), BLW, litter size, and duration of lactation (*P* < 0.05), while showing positive correlations with other indicators. WLS had no significant correlation with gestation days, temperature on the 7th day of lactation (L.d7T), temperature on the 21st day of lactation (L.d21T), L.IDF, L.IDF/SDF, and L.TDF (*P* > 0.05). It had negative correlations with parity, temperature on the 1st day of lactation (L.d1T), L.d1H, humidity on the 7th day of lactation (L.d7H), humidity on the 21st day of lactation (L.d21H), ADF content of lactation feed (L.ADF), NDF content of lactation feed (L.NDF), and SDF content of lactation feed (L.SDF), and positive correlations with the remaining indicators (*P* < 0.05). DMm and Nm had negative correlations with L.d1H, L.DM, L.Ash, L.CP, L.EE, L.P, L.Ca, L.ME, L.IDF/SDF, BLW, litter size, and duration of lactation (*P* < 0.05).

### 3.2 Feature selection results

**Fig. 4:**
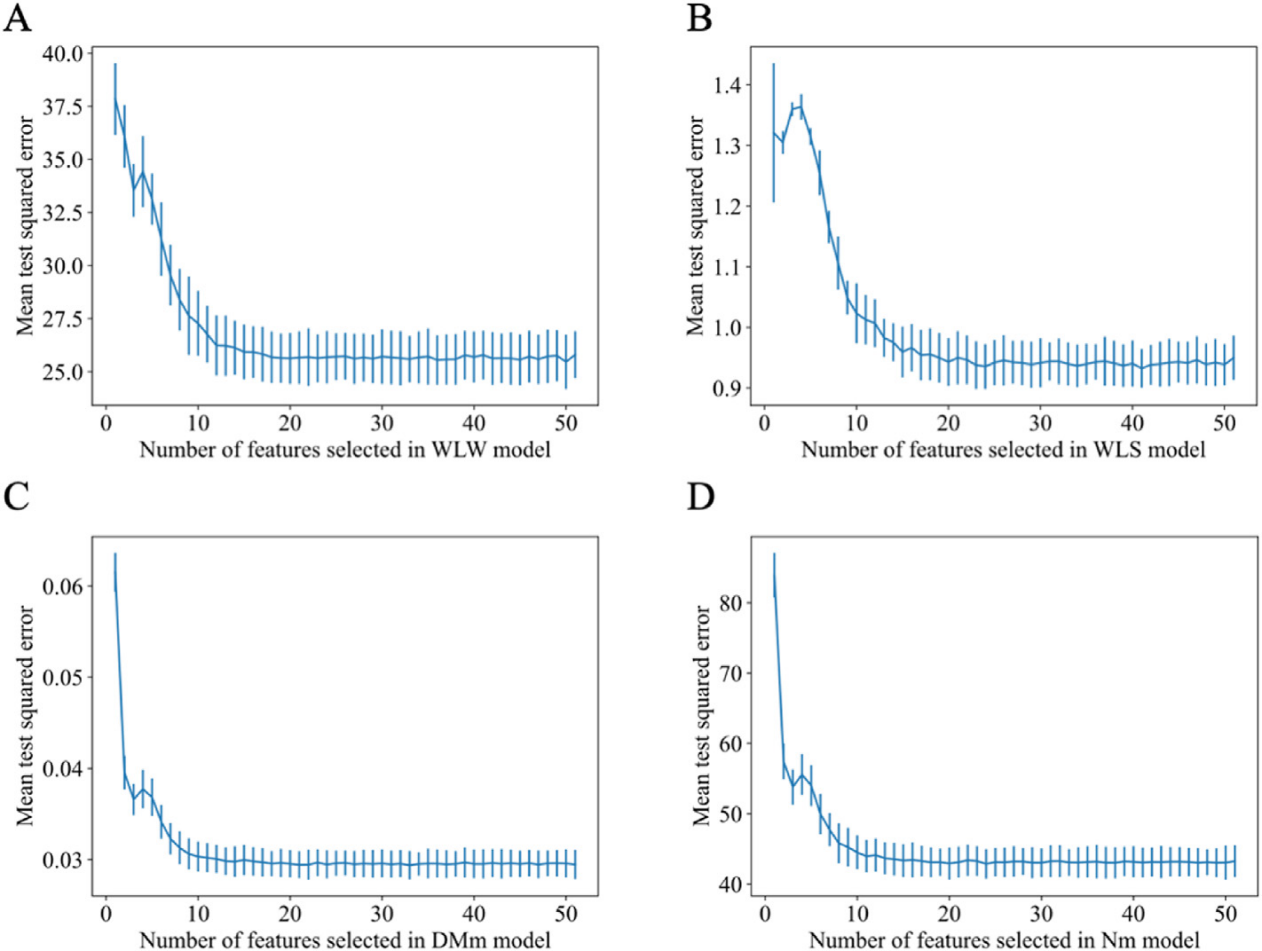
Number of features selected after applying recursive feature elimination with cross-validation (RFECV). The optimal number of features for each model are as follows: (A) WLW, (B) WLS, (C) DMm, and (D) Nm. WLW = weaned litter weight; WLS = weaned litter size; DMm = dry matter in milk; Nm = nitrogen in milk.

Figure 4 portrays the cross-validation mean squared error (MSE) of RFECV. A discernible trend emerged wherein, with an escalating number of features, the cross-validated MSE exhibited an initial steep decline followed by stabilization. This pattern suggested the existence of redundant features within the complete dataset. Notably, the inflection points outlined in Fig. 4 indicated that the optimal number of features for WLW, WLS, DMm, and Nm models were 50, 41, 33, and 24, respectively. The selected feature names were corroborated in Table 4.

**Table 4:** The selected features under recursive feature elimination with cross-validation (RFECV).

Target variables^1^	The names of selected features
WLW (50)	Parity, gestation days, L.d1T, L.d1H, L.d1BF, L.d7T, L.d7H, L.d7BF, L.d21T, L.d21H, L.d21BF, L.DM, L.Ash, L.CP, L.EE, L.P, L.Ca, L.ME, L.ADF, L.NDF, L.SDF, L.IDF, L.IDF/SDF, L.TDF, birth litter weight, litter size, duration of lactation, RSS_1–5, Breeds_1–6, strain and breed of boars_1–9, 11–13.
WLS (41)	Parity, gestation days, L.d1T, L.d1H, L.d1BF, L.d7T, L.d7H, L.d7BF, L.d21T, L.d21H, L.d21BF, L.DM, L.Ash, L.CP, L.EE, L.P, L.Ca, L.ME, L.ADF, L.NDF, L.SDF, L.IDF, L.IDF/SDF, L.TDF, birth litter weight, litter size, duration of lactation, RSS_3, Breeds_1–4, 6, strain and breed of boars _1–2, 5–7, 11–13.
DMm (33)	Parity, gestation days, L.d1T, L.d1H, L.d1BF, L.d7T, L.d7H, L.d7BF, L.d21T, L.d21H, L.d21BF, L.DM, L.Ash, L.CP, L.EE, L.P, L.Ca, L.ME, L.ADF, L.NDF, L.SDF, L.IDF/SDF, L.TDF, birth litter weight, litter size, duration of lactation, Breeds_1–4, strain and breed of boars _1–2, 6.
Nm (24)	Parity, gestation days, L.d1T, L.d1H, L.d1BF, L.d7T, L.d7H, L.d7BF, L.d21T, L.d21H, L.d21BF, L.DM, L.CP, L.EE, L.ME, L.IDF/SDF, birth litter weight, litter size, duration of lactation, Breeds_3–4, strain and breed of boars_1–2, 6.

WLW = weaned litter weight; WLS = weaned litter size; DMm = dry matter in milk; Nm = nitrogen in milk; L.d1T = temperature on the 1st day of lactation; L.d7T = temperature on the 7th day of lactation; L.d21T = temperature on the 21st day of lactation; L.d1H = humidity on the 1st day of lactation; L.d7H = humidity on the 7th day of lactation; L.d21H = humidity on the 21st day of lactation; L.d1BF = backfat thickness on the 1st day of lactation; L.d7BF = backfat thickness on the 1st day of lactation; L.d21BF = backfat thickness on the 1st day of lactation; RSS = region and strain of sows; L.DM = dry matter content of lactation feed; L.Ash = ash content of lactation feed; L.CP = crude protein content of lactation feed; L.EE = ether extract content of lactation feed; L.P = phosphorus content of lactation feed; L.Ca = calcium content of lactation feed; L.NDF = neutral detergent fiber content of lactation feed; L.ADF = acid detergent fiber content of lactation feed; L.IDF = insoluble dietary fiber content of lactation feed; L.SDF = soluble dietary content of lactation feed; L.IDF/SDF = the ratio of IDF to SDF content of lactation feed; L.TDF = total dietary fiber content of lactation feed; L.ME = metabolizable energy content of lactation feed.

1 The number in parentheses represents the total number of selected features under RFECV.

### 3.3 Model performance of different ML algorithms

**Fig. 5:**
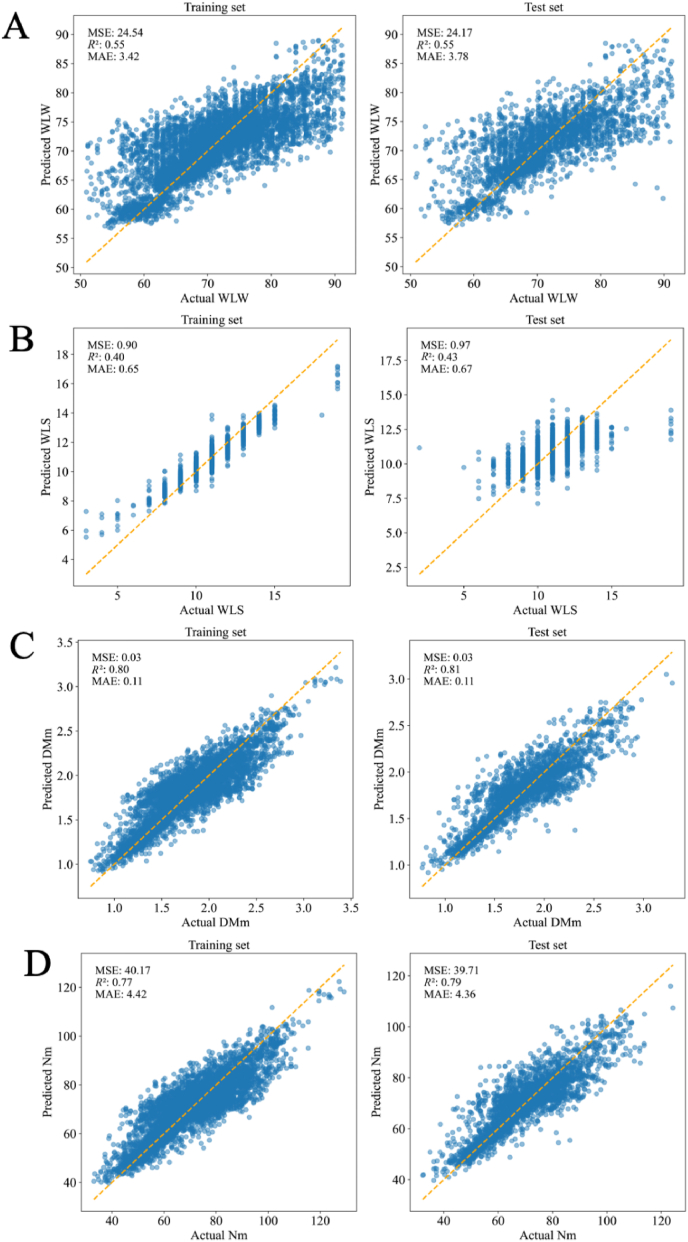
The scatter plots of the optimal algorithms for the four target variables. (A) WLW. (B) WLS. (C) DMm. (D) Nm. Left: training set. Right: test set. The orange dashed line represents the linear models where y equals x. MSE = mean squared error; MAE = mean absolute error; WLW = weaned litter weight; WLS = weaned litter size; DMm = dry matter in milk; Nm = nitrogen in milk.

On the training set, the optimal algorithm was fine-tuned using 5-fold cross-validation to determine the best parameter combination. This optimal combination was then applied to the test set to evaluate the model's performance. The optimal parameters of the trained model are presented in Table 5. Table 6 presented the *R*^2^ and MAE values from the 5-fold cross-validation on the training set alongside the *R*^2^ and MAE results on the test set. The similarity in these evaluation metrics between the training and test sets indicated a lack of overfitting. The best algorithms for predicting WLW, WLS, DMm, and Nm were GBDT, RF, GBDT, and RF, respectively. Their scatter plots are shown in Fig. 5.

**Table 5:** The optimal parameters of the trained model.

Best parameters	WLW	WLS	DMm	Nm
LR	fit_intercept: True	fit_intercept: False	fit_intercept: True	fit_intercept: True
Ridge	alpha: 0.1	alpha: 0.1	alpha: 0.1	alpha: 0.1
Lasso	alpha: 0.1	alpha: 0.1	alpha: 0.1	alpha: 0.1
ENR	alpha: 0.1, l1_ratio: 0.1	alpha: 0.1, l1_ratio: 0.1	alpha: 0.1, l1_ratio: 0.1	alpha: 0.1, l1_ratio: 0.7
SVR	C: 10, kernel: linear	C: 1, kernel: linear	C: 10, kernel: poly	C: 10, kernel: linear
DT	max_depth: 5	n_neighbors: 7	n_neighbors: 7	n_neighbors: 7
KNN	n_neighbors: 7	max_depth: 5	max_depth: 5	max_depth: 5
RF	max_depth: 10, n_estimators: 300	max_depth: 10, n_estimators: 200	max_depth: 10, n_estimators: 200	max_depth: 10, n_estimators: 300
GBDT	max_depth: 5, n_estimators: 200	max_depth: 5, n_estimators: 100	max_depth: 5, n_estimators: 200	max_depth: 5, n_estimators: 300
XGBoost	max_depth: 3, n_estimators: 300	max_depth: 3, n_estimators: 100	max_depth: 5, n_estimators: 100	max_depth: 3, n_estimators: 200
MLP	activation: tanh, hidden_layer_sizes: (50, 50)	activation: tanh, hidden_layer_sizes: (100)	activation: tanh, hidden_layer_sizes: (50, 50)	activation: relu, hidden_layer_sizes: (50, 50)

WLW = weaned litter weight; WLS = weaned litter size; DMm = dry matter in milk; Nm = nitrogen in milk; LR = linear regression; Ridge = ridge regression; Lasso = lasso regression; ENR = elasticnet regression; SVR = support vector regression; KNN = k-nearest neighbors; DT = decision tree; RF = random forest; GBDT = gradient boosting decision tree; XGBoost = extreme gradient boosting; MLP = multi-layer perceptron.

**Table 6:** The *R*^2^ and the MAE values of different machine learning algorithms on training set and test set.

Target variable	Algorithm name	Training set		Test set	
		MAE	*R*^2^	MAE	*R*^2^
WLW	LR	4.05	0.43	4.05	0.43
	Ridge	4.07	0.43	4.08	0.43
	Lasso	4.24	0.39	4.24	0.38
	ENR	4.25	0.39	4.26	0.39
	SVR	4.08	0.41	4.20	0.39
	KNN	3.64	0.50	3.57	0.52
	DT	3.86	0.46	3.80	0.48
	RF	3.50	0.54	3.45	0.54
	GBDT	3.43	0.55	3.38	0.55
	XGBoost	3.48	0.54	3.45	0.55
	MLP	3.93	0.35	3.77	0.48
WLS	LR	0.71	0.30	0.73	0.33
	Ridge	0.72	0.29	0.74	0.32
	Lasso	0.78	0.19	0.82	0.21
	ENR	0.76	0.24	0.79	0.27
	SVR	0.70	0.27	0.75	0.28
	KNN	0.69	0.30	0.71	0.36
	DT	0.71	0.31	0.73	0.35
	RF	0.65	0.40	0.67	0.43
	GBDT	0.66	0.40	0.68	0.43
	XGBoost	0.66	0.39	0.68	0.44
	MLP	0.72	0.29	0.75	0.33
DMm	LR	0.14	0.73	0.14	0.74
	Ridge	0.14	0.73	0.14	0.74
	Lasso	0.17	0.66	0.17	0.67
	ENR	0.15	0.71	0.15	0.72
	SVR	0.13	0.75	0.13	0.76
	KNN	0.13	0.75	0.12	0.78
	DT	0.14	0.73	0.14	0.74
	RF	0.12	0.79	0.12	0.80
	GBDT	0.11	0.80	0.11	0.81
	XGBoost	0.11	0.80	0.11	0.81
	MLP	0.14	0.75	0.14	0.75
Nm	LR	5.32	0.71	5.37	0.71
	Ridge	5.34	0.70	5.38	0.71
	Lasso	5.49	0.69	5.53	0.70
	ENR	5.51	0.69	5.54	0.70
	SVR	5.30	0.69	5.37	0.70
	KNN	4.92	0.72	4.83	0.75
	DT	5.30	0.70	5.39	0.71
	RF	4.57	0.76	4.52	0.78
	GBDT	4.42	0.77	4.36	0.79
	XGBoost	4.47	0.77	4.46	0.78
	MLP	5.33	0.70	5.28	0.72

WLW = weaned litter weight; WLS = weaned litter size; DMm = dry matter in milk; Nm = nitrogen in milk; *R*^2^ = coefficient of determination; MAE = mean absolute error; LR = linear regression; Ridge = ridge regression; Lasso = lasso regression; ENR = elasticnet regression; SVR = support vector regression; KNN = k-nearest neighbors; DT = decision tree; RF = random forest; GBDT = gradient boosting decision tree; XGBoost = extreme gradient boosting; MLP = multi-layer perceptron.

### 3.4 Model interpretation

**Fig. 6:**
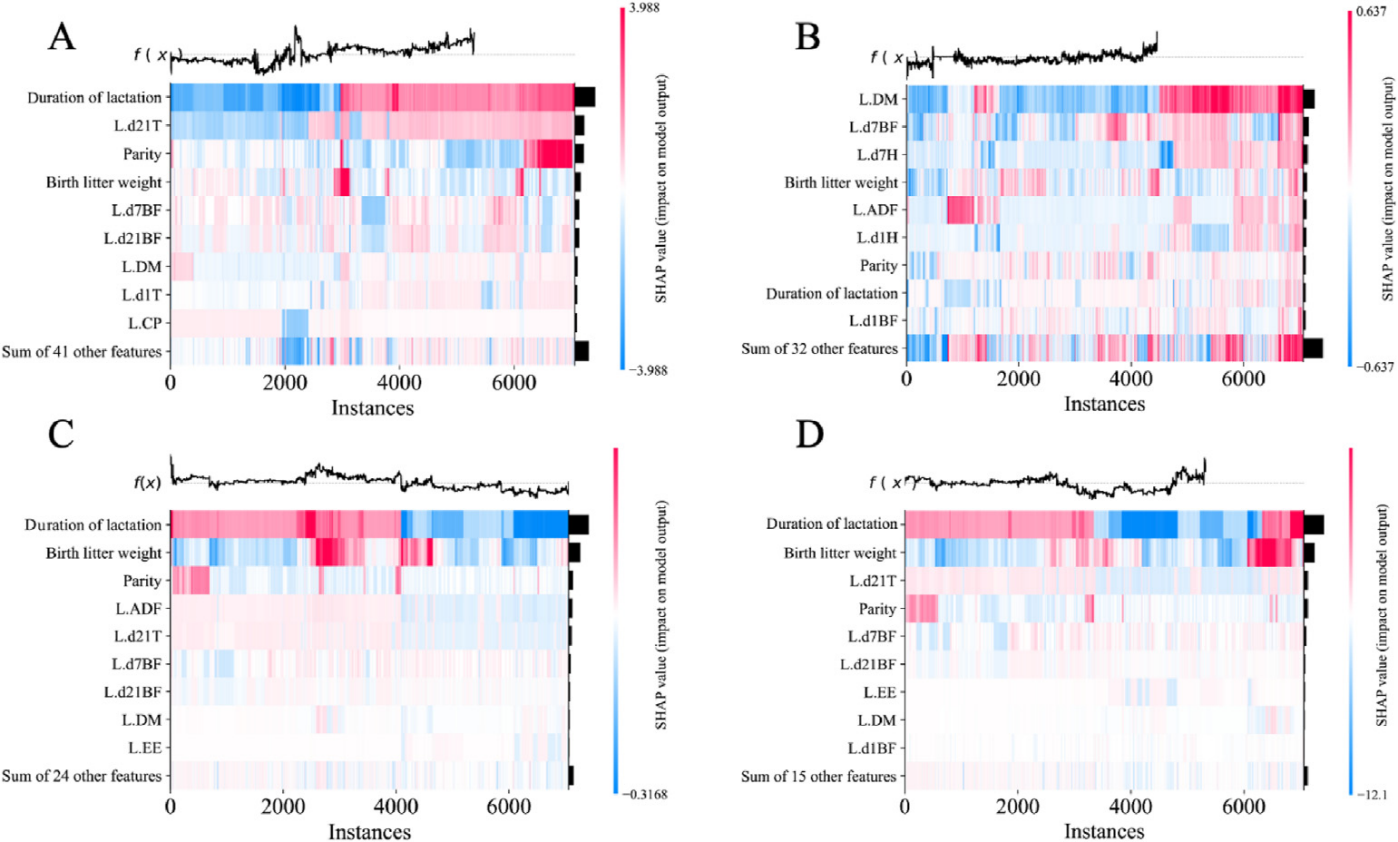
Shapley additive explanations (SHAP) heatmap of the WLW, WLS, DMm and Nm models. (A) WLW. (B) WLS. (C) DMm. (D) Nm. Instances were represented on the x-axis, model inputed on the y-axis, and SHAP values were depicted using a color scale. By default the samples were ordered based on a hierarchical clustering by their explanation similarity. This resulted in samples that have the same model output for the same reason getting grouped together. The output of the model was shown above the heatmap matrix, and the global importance of each model input shown as a bar plot on the right hand side of the plot. WLW = weaned litter weight; WLS = weaned litter size; DMm = dry matter in milk; Nm = nitrogen in milk; L.d21T = temperature on the 21st day of lactation; L.d7BF = backfat thickness on the 1st day of lactation; L.d21BF = backfat thickness on the 1st day of lactation; L.DM = dry matter content of lactation feed; L.d1T = temperature on the 1st day of lactation; L.CP = crude protein content of lactation feed; L.d7H = humidity on the 7th day of lactation; L.ADF = acid detergent fiber content of lactation feed; L.d1H = humidity on the 1st day of lactation; L.d1BF = backfat thickness on the 1st day of lactation; L.EE = ether extract content of lactation feed.

To delve deeper into the assessment of feature contributions to prediction, a matrix of SHAP values was passed to the heatmap plot function to generate a visualization. Instances were ordered by hierarchical clustering based on explanation similarity. Model output is displayed above the heatmap matrix, and input importance is shown as a bar plot on the right side (Fig. 6). For the WLW model, the top five features ranked in order of importance were duration of lactation, L.d21T, parity, BLW, and L.d7BF. For the WLS model, they were L.DM, L.d7BF, L.d7H, BLW, and L.ADF. For the DMm model, they were duration of lactation, BLW, parity, L.ADF, and L.d21T. For the Nm model, they were duration of lactation, BLW, L.d21T, parity, and L.d7BF.

## 4 Discussion

### 4.1 Feasibility of WLW, WLS, DMm and Nm prediction for precision nutrition and intelligent feeding

The best models explained over 55% of the variability in WLW, 40% in WLS, 80% in DMm, and 77% in Nm, respectively. It suggested that a significant portion of the variability in the WLW, WLS, DMm and Nm has been captured and accounted for by the model. This level of explanation indicated that the model was relatively robust and effective in predicting or understanding factors influencing the WLW, WLS, DMm and Nm of sows. Previous studies in ML related to pig production primarily focus on gene selection for predicting traits, such as litter size, feed efficiency, and growth rate (Tusell et al., 2020, 2013). Additionally, there are studies on identifying pig pathologies and predicting body weight in growing pigs, as well as investigating growth and carcass traits (Maltecca et al., 2019; Sanchez-Vazquez et al., 2012; Wang et al., 2022). However, there is a notable absence of research regarding WLW, WLS, DMm and Nm in sows. Through this research, it was evident that there was promising feasibility for predicting WLW and WLS in precision nutrition and intelligent feeding. The DMm and Nm predictions offer a practical, low-cost solution for estimating nutrient output in milk, addressing the challenge of determining nutrient requirements, which typically rely on numerous variables—such as sow and piglet weights—that are difficult to measure with current farming technologies (Gauthier et al., 2022b). Utilizing data analytics, producers can analyze historical records of sow performance, and integrate them with past nutritional and management data to build a tailored model for their swine operations. These models can be used to assess whether future management and nutrition practices contribute to improved reproductive performance and nutrient output in milk, which might enhance the sustainability and profitability of swine production operations.

### 4.2 Comparison of ML algorithms BLW prediction

Notably, comparable performance was observed between the cross-validated training results and the test results, indicating that the stratified sampling method used for data splitting was appropriate. The advantages of stratified sampling lie in its ability to significantly enhance the precision of performance assessment (Cochran, 1977). RFE adopts a recursive approach during feature selection, iteratively eliminating the least important features until the specified number of features is reached (Ramezan, 2022). This ability of RFE to comprehensively consider the information within the dataset enhances the performance of the model.

In this study, the overall performance of nonlinear ML techniques in forecasting WLW, WLS, DMm, and Nm surpassed traditional linear methods (such as LR, Ridge, Lasso, and ENR), which aligns with previous observations made from analogous analyses conducted in different domains (Cai et al., 2019; Kumar and Sivanesan, 2005). Among the four linear-based models, LR exhibited consistent yet relatively low performance across training and test sets, suggesting that it did not overfit. Therefore, optimizing the objective function with L1/L2 regularization did not lead to improved prediction accuracy. However, LR, Ridge, Lasso, ENR, and DT are more straightforward to execute and interpret. Outputs in formula form (for linear-based models) or decision tree visualizations (for DT) can offer intuitive insights into feature importance (Ruan et al., 2022).

Previous research in other domains using SVR and KNN consistently found that SVR outperformed KNN due to KNN's sensitivity to redundant and irrelevant features (Kamir et al., 2020). However, current results ran counter to this trend, with KNN overall outperforming SVR. It's possible that in current dataset, the features were structured in a way that favored the performance of KNN over SVR, perhaps due to the presence of certain patterns or relationships that KNN was better able to capture. The poor performance of the MLP may be attributed to improper selection and adjustment of hyperparameters, requiring further tuning. RF, GBDT and XGBoost demonstrated superior performance over DT for prediction, as they can handle high-dimensional datasets and avoid overfitting (Chen et al., 2020; Ruan et al., 2022). Given that there is no universally superior algorithm, the most appropriate choice depends on the specific task and dataset (Haraty and Assaf, 2024). With this in mind, ensemble learning algorithms emerge as the most promising option for predicting WLW, WLS, DMm, and Nm in our dataset.

### 4.3 The analysis of contributory features of the WLW, WLS, DMm, and Nm models

WLW is a crucial indicator of the growth, development, and health status of nursing piglets. The quality and quantity of milk have a significant impact on WLW (Koketsu and Dial, 1997). This relationship is further evidenced by correlation analysis, which shows both DMm and Nm have correlations greater than 0.8 with WLW. However, L.d7H and L.d7BF exhibited little correlation with the four target variables in the Spearman correlation analysis, yet they ranked among the top five important features in the models. This discrepancy could be due to the fact that correlation analysis does not capture all the complexities that ML models can handle, such as non-linear relationships, interactions, and multicollinearity (Murdoch et al., 2019). The prominence of L.d7H and L.d7BF in the models could be attributed to the increased milk demand piglets experience during their first week of life. When sows are exposed to unfavorable environmental conditions, their feed intake decreases, leading to reduced backfat thickness and insufficient milk production during this critical period. This, in turn, may compromise piglet survival due to inadequate nutrition (Wegner et al., 2016).

The global importance of each model input, as revealed by SHAP analysis, provides insights into the features that have the most significant influence on the variability of WLW, WLS, DMm, and Nm. It is well established that the quality and quantity of milk are closely linked to the sow's nutrient intake and management practices during the gestation period (Kim, 2013). These factors are also reflected in BLW, which explains why BLW is among the top five important factors for WLW, WLS, DMm, and Nm. DMm and Nm represent the dry matter and nitrogen content of milk produced by each sow throughout the entire lactation period. Thus, their strong correlation with the duration of lactation is expected. However, the negative correlation could be due to several factors. It may be related to the physiological changes in sows during the lactation period, where prolonged lactation could lead to decreased nutritional efficiency or milk production capacity. Additionally, fluctuations in nutritional demands and stress levels over time may affect the composition of the milk (Chisoro et al., 2023). Moreover, parity is identified as a key influencing factor for all four target variables, consistent with previous studies showing that parity significantly impacts reproductive performance (Knecht et al., 2015). Reproductive efficiency generally improves with increasing parity, with the peak reproductive performance typically observed between parity 3 and 5, coinciding with changes in parity at weaning (Hoving et al., 2011).

It is widely known that milk production accounts for 65% to 80% of the energy requirements of lactating sows (NRC, 2012). Energy intake is typically lower than lactation demands, resulting in a negative energy balance for sows during most of the lactation period (NRC, 2012). Feed intake and nutrition during lactation play a crucial role in determining the quality and quantity of milk (Sulabo et al., 2010b). Unfortunately, due to substantial missing data on sow feed intake during lactation in this experiment, feed intake data was excluded from the analysis. Moving forward, collecting more feed intake data from pig farms will be essential to gain deeper insights into the factors affecting WLW, WLS, DMm, and Nm. Backfat thickness is a key indicator of maternal nutritional status during lactation. Across the four models, backfat thickness was found to significantly impact variability in the target variables. Since milk production is prioritized during lactation, sows will mobilize body tissues to sustain milk output, leading to weight loss (Strathe et al., 2017). This presents an opportunity to develop nutritional strategies to stimulate sows to achieve an optimal level of energy consumption with minimal mobilization of body reserves (Tokach et al., 2019).

### 4.4 Potential of the WLW, WLS, DMm, and Nm prediction model

The WLW, WLS, DMm, and Nm prediction model represents a groundbreaking advancement in the field of swine husbandry, particularly in improving sow reproductive performance and nutrient output in milk. By leveraging advanced algorithms and comprehensive datasets that encompass a variety of environmental and nutritional factors, these models offer unparalleled insights into the potential WLW, WLS, DMm, and Nm of sows. They have the potential to revolutionize decision-making processes within the swine industry, enabling stakeholders to make informed choices regarding breeding practices, nutrition management, and resource allocation.

Looking ahead, the integration of the WLW, WLS, DMm, and Nm prediction models with the Internet of Things (IoT) and smart livestock management systems opens exciting new possibilities for the future of swine production (Su et al., 2024). With advancements in sensor technology, including wearable devices and implantable sensors, coupled with sophisticated analytics powered by artificial intelligence and ML algorithms, farmers will gain unprecedented insights into sow reproductive performance, nutrient output in milk and nutritional needs (Zhang et al., 2021), making informed decisions regarding precision nutrition. This enhanced level of data-driven decision-making will not only drive improvements in productivity and efficiency but also elevate animal welfare standards and environmental sustainability within the industry. Moreover, the interconnected nature of these technologies will facilitate seamless collaboration and information sharing across the entire swine supply chain, from farm to fork.

## 5 Conclusion

In this study, comprehensive models were developed to predict WLW, WLS, DMm, and Nm based on various features related to swine nutrition and environmental conditions. These features can be easily collected on-farm and effectively integrated into our models. Current findings indicate that ensemble learning methods, such as RF and GBDT, deliver the most effective performance. Looking ahead, the integration of these models with intelligent feeding systems holds the potential to transform swine management by enabling tailored feeding strategies that ensure precision nutrition, boost production efficiency, enhance animal health and welfare, and reduce environmental impact.

## Credit Author Statement

**Jiayi Su:** Writing – original draft, Visualization, Methodology, Formal analysis. **Xiangfeng Kong:** Supervision. **Wenliang Wang:** Writing – original draft. **Qian Xie:** Data curation. **Chengming Wang:** Data curation. **Bie Tan:** Project administration, Funding acquisition. **Jing Wang:** Supervision, Project administration, Funding acquisition.

## Declaration of competing interest

We declare that we have no financial and personal relationships with other people or organizations that can inappropriately influence our work, and there is no professional or other personal interest of any nature or kind in any product, service and/or company that could be construed as influencing the content of this paper.
